# Supplementary material for: The causal relationship between the human gut microbiota and pyogenic arthritis: a Mendelian randomization study
Source: Front Cell Infect Microbiol. 2024 Nov 26;14:1452480. doi: 10.3389/fcimb.2024.1452480 (PMC11629706; doi:10.3389/fcimb.2024.1452480)
Supplement: Supplementary file 1 [file DataSheet1.zip › mendelian test/ebi-a-GCST90027786.csv_forest.pdf]

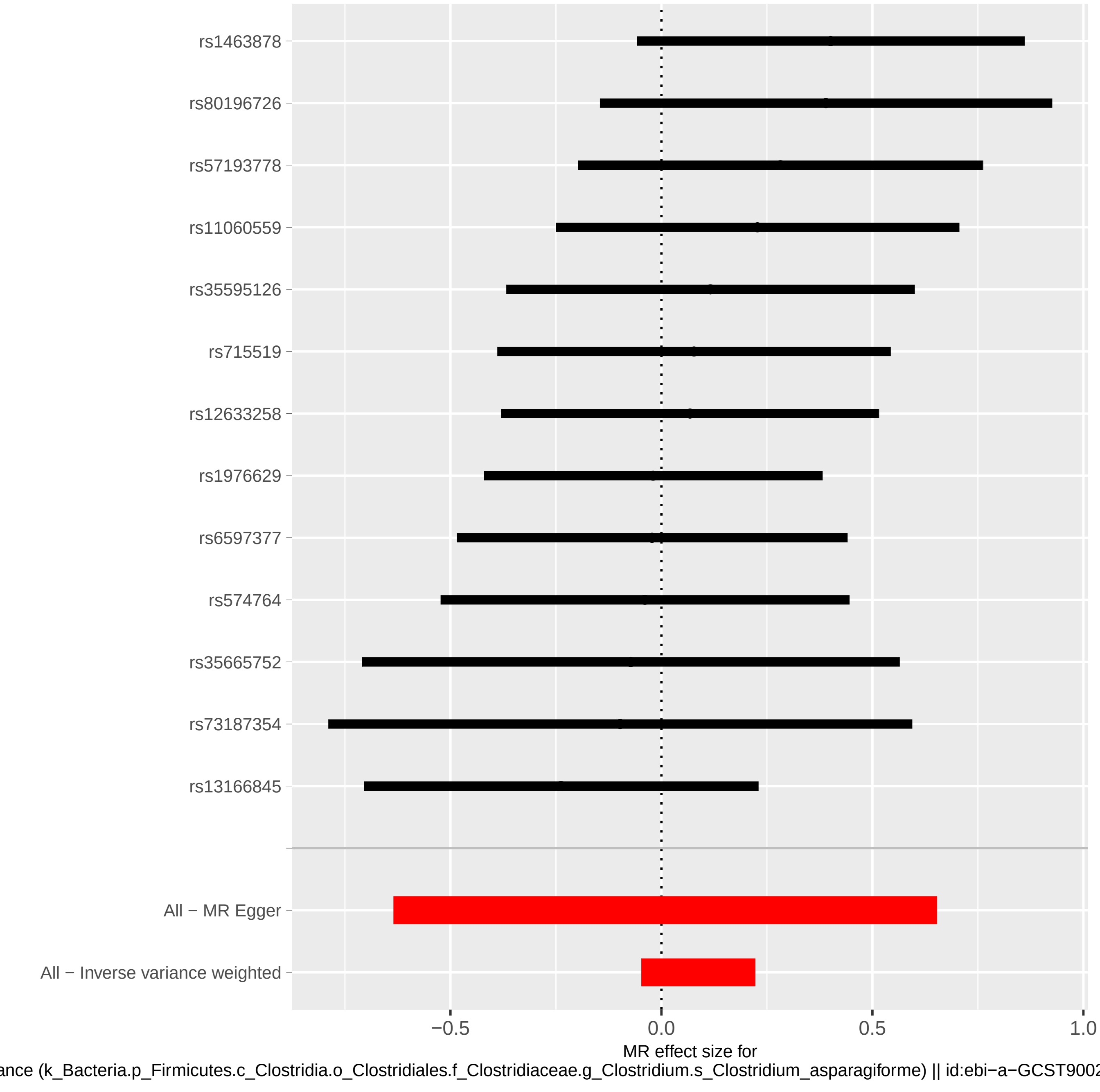
rs1463878
rs80196726
rs57193778
rs11060559
rs35595126
rs715519
rs12633258
rs1976629
rs6597377
rs574764
rs35665752
rs73187354
rs13166845
All − MR Egger
All − Inverse variance weighted
−0.5
0.0
0.5
1.0
MR effect size for
ance (k_Bacteria.p_Firmicutes.c_Clostridia.o_Clostridiales.f_Clostridiaceae.g_Clostridium.s_Clostridium_asparagiforme) || id:ebi−a−GCST9002
